# Differential effects of temporal regularity on auditory-evoked response amplitude: a decrease in silence and increase in noise

**DOI:** 10.1186/1744-9081-9-44

**Published:** 2013-12-03

**Authors:** Hidehiko Okamoto, Henning Teismann, Sumru Keceli, Christo Pantev, Ryusuke Kakigi

**Affiliations:** 1 Department of Integrative Physiology, National Institute for Physiological Sciences, 38 Nishigo-Naka, Myodaiji, Okazaki 444-8585, JAPAN; 2 Institute for Biomagnetism and Biosignalanalysis, University of Muenster, Muenster, Germany; 3 Institute for Epidemiology and Social Medicine, University of Muenster, Muenster, Germany

**Keywords:** Auditory cortex, Auditory scene analysis, Human, Magnetoencephalography, N1m

## Abstract

**Background:**

In daily life, we are continuously exposed to temporally regular and irregular sounds. Previous studies have demonstrated that the temporal regularity of sound sequences influences neural activity. However, it remains unresolved how temporal regularity affects neural activity in noisy environments, when attention of the listener is not focused on the sound input.

**Methods:**

In the present study, using magnetoencephalography we investigated the effects of temporal regularity in sound signal sequencing (regular vs. irregular) in silent versus noisy environments during distracted listening.

**Results:**

The results demonstrated that temporal regularity differentially affected the auditory-evoked N1m response depending on the background acoustic environment: the N1m amplitudes elicited by the temporally regular sounds were smaller in silence and larger in noise than those elicited by the temporally irregular sounds.

**Conclusions:**

Our results indicate that the human auditory system is able to involuntarily utilize temporal regularity in sound signals to modulate the neural activity in the auditory cortex in accordance with the surrounding acoustic environment.

## Background

We are continuously exposed to environmental sounds in daily life. Task-relevant sound signals are often hidden in irrelevant noise. However, even when we do not voluntarily focus on surrounding sounds, we can easily detect such relevant sound signals despite the presence of ambient noises (e.g., someone calling our name in a noisy environment). Therefore, it seems plausible that humans are capable of continuously and involuntarily monitoring and segregating their acoustic environment [1]. However, the neural mechanisms that enable this accomplishment even when attention is not focused on the auditory input still remain elusive.

Previous studies have shown that the neural responses evoked by sounds in noisy conditions differ from those evoked during silent conditions. Auditory-evoked responses were shown to be reduced and delayed in noisy environments [2-4]. On the other hand, Alain *et al.*[5] showed that low intensity background noise could enhance the amplitude of auditory responses evoked by sound signals. These results indicated that background noise could lead to both a decrease and increase in the auditory-evoked response amplitude, depending on the situation during which the sound signals appear.

The repetition of identical sound stimuli in a silent background is known to lead to decreased N1(m) responses ([6,7], for a review see [8]). However, a previous study [9] demonstrated that the repetition of a constant frequency sound in noisy environments resulted in significantly larger N1m responses than those elicited by randomly presented frequencies, even when the participants did not pay attention to the auditory modality. These results support the hypothesis that predictable auditory patterns aid the perception of the auditory scene [10] while attention is not focused on the auditory signals. The auditory neural pathway is tonotopically organized [11-13]. In noisy environments, spectral cues appear to enhance the neural activity located at the corresponding tonotopic map spot in the human auditory neural pathway even when the listener’s attention is distracted from the auditory input. In the study described above [9], all test sounds were presented in a temporally regular manner (inter stimulus interval (ISI) = 2400 ms). Listeners implicitly knew the timing and the frequencies of the upcoming sound stimuli in the constant frequency sound sequence; therefore, they could involuntarily assign their processing resources to the auditory neurons corresponding to these frequencies in a timely manner, resulting in larger amplitudes and shorter latencies of the auditory-evoked responses. Therefore, we assume that predictable auditory patterns may not be limited to spectral information; in noisy environments, temporal information may also play an important role for the auditory neural processing.

Based on these considerations, the goal of the present study was to investigate the effects of temporal regularity in sound signals on auditory-evoked responses in both silent and noisy environments while the participants’ attention was distracted from the auditory modality. A regular ISI may enable participants to involuntarily modulate their neural activity in the time domain to the appearance or absence of the sound signals, while that would be difficult with irregular ISIs. We hypothesized that even in an unattended situation the effects of temporal regularity on the auditory-evoked responses would differ between silence and noisy environments, in which the listeners would have to segregate sound signals from ambient noise.

## Methods

### Participants

Thirteen healthy participants (5 females, age range 23 – 44 years) participated in the present study. All participants had normal hearing and no neurological disorders. All participants were fully informed about the study and gave written informed consent for their participation in accordance with procedures approved by the Ethics Commission of the National Institute for Physiological Sciences. The study thus conformed to The Code of Ethics of the World Medical Association (Declaration of Helsinki).

### Stimuli and experimental design

**Figure 1:**
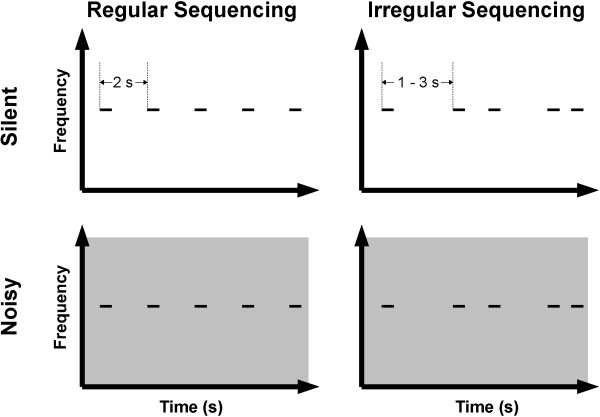
**Experimental design.** Schematic display of auditory stimulation in the regular sequencing (left column) and irregular sequencing conditions (right column), and in the silent (upper panels) and noisy (lower panels) conditions. The test stimulus (TS) and background noise are represented by short black solid lines and gray areas, respectively. Sound onset asynchrony between two successive TS was 2 s in the regular sequencing condition (left column), and either 1.0, 1.5, 2.0, 2.5, or 3.0 s in the irregular sequencing condition (right column).

**Figure 2:**
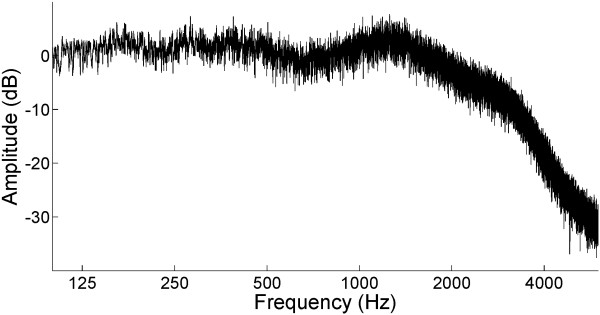
**The amplitude spectrum of white noise recorded at the ear piece.** Because of our sound delivery system (plastic tube and ear piece), the sound spectrum exhibited a 2 kHz low-pass filtered characteristic. Sample audio files of the test stimulus embedded in the background noise are available as Additional files 1 (Regular Sequencing) and 2 (Irregular Sequencing).

The experimental design is schematically represented in Figure 1. The test stimulus (TS) was a 1000 Hz pure tone. The TS was presented either together with broad-band background noise or in silence. Sound onset asynchrony was fixed to 2000 ms in the regular-sequencing condition, or pseudo-randomly selected from 1000, 1500, 2000, 2500, and 3000 ms in the irregular-sequencing condition (Figure 1). The TS had a fixed duration of 500 ms, including 10 ms onset- and offset-ramps. Therefore, the inter-stimulus interval (ISI) between the offset of a TS and the onset of the following TS was either regular (1500 ms) or irregular (500, 1000, 1500, 2000, or 2500 ms). All sounds were diotically presented through plastic tubes of 1.5 m length and earpieces fitted to the participant’s ears. The noise recorded at the earpiece using an ear simulator (Type 4157, Brüel & Kjaer Sound & Vibration Measurement, Naerum, Denmark) showed a low-pass filtered frequency characteristic, reflecting the frequency response of our sound delivery system (Figure 2). Before starting magnetoencephalography (MEG) data acquisition, each participant’s hearing threshold for the TS was individually determined for each ear. During the MEG recording session, the 1000 Hz TS was presented at an intensity of 40 dB above the individual sensation level. The broadband masking noise had 10 dB more power (not loudness) than the TS (cf. Additional file 1 (audio file: regular sequencing in noise) and Additional file 2 (audio file: irregular sequencing in noise)). In order to keep the test participants alert and distracted from the auditory signals, a self-chosen silent movie was presented during the MEG recordings. At the end of the measurement, questions regarding the content of the movie were asked to ensure that the participants had paid attention to the movie.

In order to investigate temporal regularity (Regular vs. Irregular) and noise level (Silent vs. Noisy) effects, we used four different conditions (Figure 1): regular sequencing in silence (Regular_Silent), irregular sequencing in silence (Irregular_Silent), regular sequencing in noise (Regular_Noisy), and irregular sequencing in noise (Irregular_Noisy). Each MEG session consisted of eight blocks (two blocks per condition) of 150 trials, resulting in 300 trials per condition. The block order was pseudo-randomized among participants. The mean ISI of the irregular-sequencing condition was kept equal to the mean ISI of the regular-sequencing condition (mean ISI = 1500 ms).

### Data acquisition and analysis

Auditory-evoked fields were recorded with a helmet-shaped, 306 channel MEG system (Vector-view, ELEKTA, Neuromag, Helsinki, Finland) with 102 identical triple sensor elements located in a silent, magnetically shielded room. We analyzed the MEG signals recorded by 204 planar-type gradiometers, and detected the largest signals over the corresponding cerebral sources. Signals were passed through a 0.03 – 200 Hz band-pass filter and digitized at 600 Hz. The magnetic fields evoked by TS were averaged selectively for each condition, starting 300 ms prior to TS onset, and ending 200 ms after TS offset. Participants were instructed not to move their heads during the recordings; compliance was monitored through a video camera by the experimenter. Epochs containing amplitude changes greater than 3 pT were discarded as artifact-contaminated epochs.

The locations and orientations of the equivalent current dipoles were estimated using the BESA software (BESA Research 5.3.7, BESA GmbH, Germany). To analyze the N1m component, which is the major deflection of the auditory-evoked field (for reviews see [8,14]), the averaged fields were 30 Hz low-pass filtered (zero-phase shift Butterworth filter, 24 dB/oct), and the baseline was corrected relative to the 250 ms pre-stimulus interval. Previous studies [2,4,15-17] showed that the calculated source locations and orientations of the N1m responses were not influenced by the presence of acoustic noise. Moreover, the estimated single dipole source strength was shown to be dependent on the depth of the estimated location [18]. Thus, in order to improve the signal-to-noise ratio, we grand-averaged the magnetic fields of all conditions and used these grand-averaged magnetic waveforms to estimate the single equivalent current dipoles reflecting the N1m response. The peak N1m response was initially identified as the maximal root-mean square value of the global field power around 100 ms after TS onset. The 10 ms time window around the peak was then used for dipole source estimation. Source locations and orientations were then estimated at the N1m amplitude peak by means of single equivalent current dipole modeling (one dipole per hemisphere) for each participant individually. Estimated sources, which were fixed in location and orientation for each hemisphere of each participant, served as a spatial filter [19] during the calculation of the source strength waveforms for each condition. The mean source strength within the 10 ms time window around the peak N1m latency in each hemisphere and each condition in the time range between 80 and 300 ms was used for statistical analysis.

In order to evaluate the effects of noise and temporal regularity, the source strengths and latencies of the N1m responses averaged across hemispheres in each condition were analyzed separately via a repeated-measures analysis of variance (ANOVA) using the two factors NOISE_LEVEL (Silent vs. Noisy) and SEQUENCING (Regular vs. Irregular).

## Results

**Figure 3:**
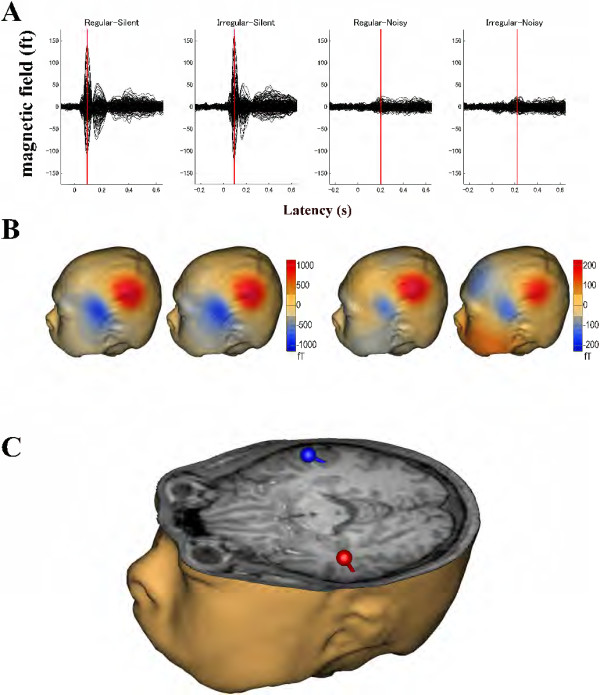
**Representative subject result. (A)** Individual auditory-evoked magnetic fields under each condition. N1m responses are indicated by the red vertical lines. **(B)** Isocontour maps of the magnetic fields at the N1m latency. The magnetic contour maps show clear dipolar patterns above the left auditory cortex. Red and blue contour lines represent the outbound and inbound flows of magnetic fields from and into the brain. Different scales were used between the silent and noisy conditions. **(C)** Source estimation. Estimated equivalent current dipoles at the latency of the maximal N1m response are illustrated together with a three-dimensional head and brain model reconstructed from the individual MRI. The spheres and barrels indicate the locations and orientations of the single dipoles in the left (red) and right (blue) hemispheres.

After artifact rejection, more than 90% of trials could be averaged for each condition in all participants (mean ± standard deviation: Regular_Silent = 298.7 ± 2.6 trials, Irregular_Silent = 297.5 ± 2.3 trials, Regular_Noisy = 296.4 ± 3.6 trials, Irregular_Noisy = 297.8 ± 3.1 trials). Dipolar magnetic field patterns over the left and right hemispheres were observed in all conditions (Figure 3A and B). The amplitudes of auditory-evoked fields were much larger in silent conditions than in noisy conditions. However, magnetic field distributions were very similar between silent and noisy conditions, indicating that the foci of the neural sources were similar. The grand-averaged waveforms used for the equivalent current dipole estimation and estimated source locations and orientations of the N1m response overlaid on the axial slice of the structural magnetic resonance image of one representative participant are displayed in Figure 3C. The goodness-of-fit of the underlying dipolar source models for the grand-averaged MEG waveforms was above 90% in all cases. The estimated dipolar sources were located at the superior temporal plane, which corresponded to the N1m generator [12,20].

**Figure 4:**
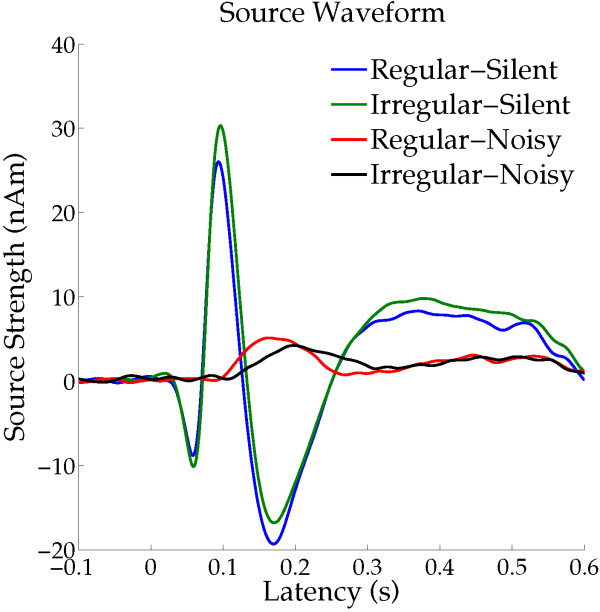
**Time courses of the mean source strengths across all participants (N = 13) and hemispheres.** Each colored line represents an experimental condition (see legends in the right upper corner).

**Figure 5:**
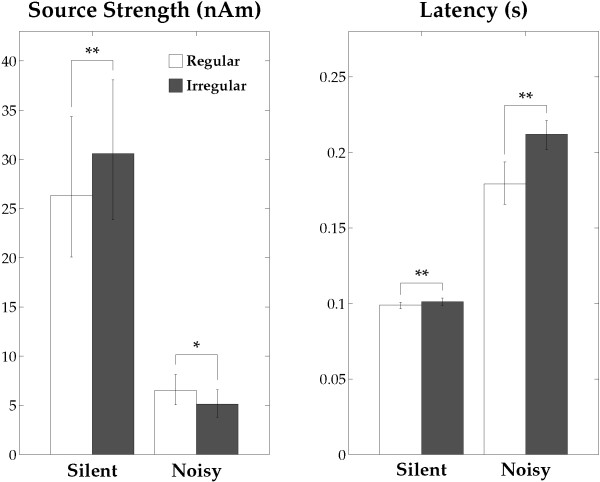
**N1m source strengths and latencies.** Group means (N = 13) of the N1m source strengths (left graph) and latencies (right graph) in the “Silent” and “Noisy” conditions including error bars denoting the 95% confidence intervals. Open and filled bars denote the “Regular” and “Irregular” conditions (* *p* < 0.05, ** *p* < 0.01 (Bonferroni-corrected)).

The time courses (time range from -100 to +650 ms) of the source strengths averaged across all participants and hemispheres are displayed in Figure 4. The N1m responses had larger amplitudes and shorter latencies overall in the silent condition than in the noisy condition. Figure 5 shows the mean N1m source strengths and latencies in each condition together with the corresponding 95% confidence intervals. Repeated-measures ANOVAs evaluating N1m source strength and N1m latency resulted in significant main effects for NOISE_LEVEL (Source strength: F (1, 12) = 39.01, *p* < 0.001; Latency: F (1, 12) = 284.86, *p* < 0.001) and SEQUENCING (Source strength: F (1, 12) = 13.81, *p* < 0.01; Latency: F (1, 12) = 47.73, *p* < 0.001). Additionally, there were significant interactions between NOISE_LEVEL and SEQUENCING (Source strength: F (1, 12) = 24.69, *p* < 0.001; Latency: F (1, 12) = 34.26, *p* < 0.001). Planned contrasts (Regular_Silent vs. Irregular_Silent; Regular_Noisy vs. Irregular_Noisy) were calculated in order to further explore the interactions between NOISE_LEVEL and SEQUENCING. The Bonferroni multiple comparison correction was used to control the family-wise error rate. The N1m source strength in the silent condition was significantly larger for irregular than for regular (two-tailed paired t-test: t(12) = 4.876, *p* < 0.001 (Bonferroni-corrected)), whereas the N1m source strength in the noisy condition was significantly larger for regular than for irregular (t(12) = 3.27, *p* < 0.02 (Bonferroni-corrected)). The N1m latency was significantly longer for irregular than for regular both in the silent (t(12) = 4.351, *p* < 0.002 (Bonferroni-corrected)) and noisy (t(12) = 6.410, *p* < 0.001 (Bonferroni-corrected)) conditions. The significant interaction between NOISE_LEVEL and SEQUENCING for the N1m source strength demonstrated that regular (compared to irregular) SEQUENCING increased neural activity under the noisy condition, while neural activity was decreased under the silent condition. Moreover, the regular sequencing shortened the N1m latency in both the silent and noisy conditions.

## Discussion

The results of the present study demonstrated that the magnitude and latency of neural responses elicited in silent and noisy environments depended on temporal regularity in the sound sequences used to evoke the responses, even when the participants did not pay attention to the auditory signals. N1m latencies were shorter with regular ISI than with irregular ISI in both the silent and noisy conditions. However, N1m response amplitudes were smaller with regular ISI than with irregular ISI in the silent condition, whereas they were larger in the noisy condition. Notably, the test stimulus sequence was identical between the silent and noisy conditions. To the best of our knowledge, these results are the first to demonstrate the differential effects of temporal regularity on auditory-evoked response amplitudes in noisy versus silent backgrounds during distracted listening.

In a silent environment, repeated exposure to sounds with identical features may induce neural adaptation and a consequent decline in the auditory-evoked response amplitude [21-23]. In the present study, participants could implicitly foresee the timing of the onset of the upcoming TS during the regular ISI condition, whereas this was difficult in the irregular sequencing condition. Previous electroencephalography and MEG studies [24-26] demonstrated that knowledge of the stimulus onset timing could reduce N1(m) amplitude and shorten N1(m) latency in silent background conditions. The present study also confirmed that N1m source strengths were smaller and N1m latencies were shorter with the regular than with the irregular condition in silence (Figures 4 and 5). In the silent environment, neural adaptation in response to stimulus timing may have led to lower N1m amplitudes and shortened N1m latencies elicited by the TS with regular ISI than with irregular ISI. Moreover, a recent functional MRI (fMRI) study [27] investigated neural activity while participants were listening to temporally regular or irregular sequences of tones and were performing an intensity discrimination task (not periodicity detection task). The authors found that in silence, the regular sequences caused larger neural activity in the putamen and smaller neural activity in the primary and secondary auditory cortices than the irregular sequences. In the present study, after regular sequences in silence, we also observed smaller N1m responses, which originate in non-primary auditory cortex; however, we were not able to study neural activity in the putamen. The reason is that the MEG-sensors are very sensitive to superficial activity originating in cortical sulci, but almost insensitive to the neural activity in the putamen. A cortical-striatal system (for reviews see [28,29]) appears to be involved in the neural processing of temporal regularity in sound sequences. Enhanced neural activity in the striatum appears to take charge of the neural processing of temporally regular sounds, resulting in reduced neural activity in the auditory cortex.

The present results observed in the noisy condition, showing larger N1m responses with the regular than with the irregular sequencing condition, seem at first sight contradictory to the results obtained both in the silent condition and in previous studies [24-26,30,31]. In contrast to the current knowledge of the neural adaptation of auditory-evoked responses in silence, very little is known for noisy environments. While participants were distracted from the auditory modality, Lagemann *et al.*[32] presented a train of four consecutive tones of the same frequency separated by a regular silent interval of 500 ms under a broadband masking environment. The authors demonstrated that the auditory-evoked field amplitudes were similar between the first, second, third, and fourth tones. Therefore, the neural mechanisms of adaptation to the sound signals might differ in silent and noisy environments. Temporal regularity in sound inputs in a noisy environment may have driven the involuntary constitution of a specific auditory stream as a figure, which may then have been easily segregated from the background noise by the listeners [1,5,33-35]. However, the formation of an auditory stream may have been unstable in case of the irregular ISI condition. The significant results observed in this study suggest that the bottom-up driven formation of auditory figure-ground segregation may facilitate neural activity tracking of the regular ISI test sound signals. Teki *et al.*[35] investigated the neural bases of auditory stimulus-driven figure-ground segregation by using a unique stimulus that incorporated stochastic variation of the signal components in frequency-time space. Figure and ground auditory signals overlapped in spectrotemporal space, but differed in their statistics of fluctuations. By means of fMRI the authors measured the brain activity related to figure-ground decomposition while the participants performed an irrelevant task. The authors observed significantly increased activations in the intraparietal sulcus, the superior temporal sulcus, and the right planum temporale as a function of increasing duration of the figures, and increased activations in the intraparietal sulcus and the superior temporal sulcus as a function of increasing the number of components of the figures. In the present study, in the Regular_Noisy condition we also observed an enlarged N1m response, which appears to originate mainly in the planum temporale [12,20,36]. Neural activity in the supra temporal sulcus might partially contribute to the increased N1m amplitude in the Regular_Noisy condition, since the dipole location merely represents the center of gravity of the neural responses, and not the extent of activated areas. On the other hand, we did not observe intraparietal sulcus activity in the Regular_Noisy condition, as shown in Figure 3. This inconsistency could be due to the different functional neuroimaging procedures (fMRI vs. MEG). In the present MEG study, we analyzed the auditory-evoked N1m response that was precisely time-locked to the bottom-up sound inputs and had a specific latency in a millisecond temporal resolution. Therefore, the neural activity in the intraparietal sulcus, which is an area outside the classical auditory cortex, might not be time-locked to the bottom-up sound inputs, or might have a different latency from the N1m response, leading to almost no contribution to the auditory-evoked N1m response measured by MEG.

In the present study, we used five different ISIs (500, 1000, 1500, 2000, or 2500 ms) in the irregular sequencing condition and only one ISI (1500 ms) in the regular condition. Longer ISIs are known to elicit larger N1m responses than shorter ISIs, and this effect was shown to be non-linear [37-41]. Therefore, even though the mean values of the irregular and regular ISIs were identical, the non-linearity of the ISI effect may have led to differential N1m response amplitudes for the irregular and regular ISI conditions. The amplitudes of the auditory-evoked responses elicited in a silent environment were shown to have a negative exponential dependence on the ISI [37,42]. This negative exponential ISI dependence may have led to smaller N1m amplitudes in the irregular sequencing condition than in the regular sequencing condition. However, the present study showed that the N1m amplitudes obtained in the silent condition were significantly larger in the irregular than in the regular condition. Therefore, the negative exponential dependence of the N1m amplitude on ISI alone cannot explain the obtained results.

## Conclusions

Our present findings demonstrated that the human auditory system is able to implicitly utilize temporal regularities in sound signals to modulate neural activity. Under circumstances permitting trouble-free sound detection, for instance in silence, the auditory system seems to be able to reduce the amount of neural activity allocated for processing temporally regular sounds. In contrast, when the circumstances are less optimal and auditory figure-ground segregation is required (e.g., in the presence of disturbing noise) the auditory system may use temporal regularity to properly allocate neural resources along the time axis and to effectively segregate a sound signal from the background.

## Abbreviations

ANOVA: Analysis of variance; fMRI: functional magnetic resonance imaging; ISI: Inter-stimulus interval; MEG: Magnetoencephalography; TS: Test stimulus.

## Competing interests

All authors declare that they have no conflicts of interests.

## Authors’ contributions

Conceived and designed the experiments: HO. Performed the experiments: HO, SK. Analyzed the data: HO, HT. Drafted the manuscript: HO. Revised the manuscript: HO, HT, SK, CP, RK. All authors read and approved the final manuscript.

## Supplementary Material

Additional file 1 An exemplary sound representing regular sequencing in the noisy condition. Click here for file

Additional file 2 An exemplary sound representing irregular sequencing in the noisy condition. Click here for file
